# Efficacy and safety of traditional Chinese medicines combined with conventional Western medicines in the treatment of type 2 diabetes mellitus: a network meta-analysis of randomized controlled trials

**DOI:** 10.3389/fendo.2023.1134297

**Published:** 2023-05-08

**Authors:** Kaile Ma, Lijuan Zhou, Yanjiao Zhang, Jingyi Zhao, Chensi Yao, Chuanxi Tian, Min Li

**Affiliations:** ^1^ Institute of Metabolic Diseases, Guang’ anmen Hospital, China Academy of Chinese Medical Sciences, Beijing, China; ^2^ Beijing University of Chinese Medicine, Beijing, China

**Keywords:** type 2 diabetes, traditional Chinese medicine, conventional western medicine, network meta-analysis, meta-analysis

## Abstract

**Aims/hypothesis:**

Through a comprehensive analysis of the clinical randomized controlled trials of traditional Chinese medicine (TCM) combined with conventional western medicine (CWM) in treating type 2 diabetes(T2DM) in the past ten years, the clinical efficacy and safety of different TCMs combined with CWM were explored. This study aimed to provide specific suggestions for clinical guidance on treating T2DM.

**Methods:**

A literature search was conducted in CNKI, WanFang, VIP, CBM, PubMed, Embase, and Web of Science. The search time was limited from 2010 to the present time. The literature type was a controlled clinical trial study of TCM combined with CWM intervention in treating T2DM. The outcome indices of the efficacy evaluation included fasting blood glucose (FBG), 2-hour postprandial blood glucose (2hPG), glycosylated hemoglobin (HbA1c), adverse reactions, and clinical efficacy. Stata 15 and RevMan 5.4 software were used to conduct a network meta-analysis and a traditional meta-analysis.

**Results:**

The results showed that shenqi jiangtang granule combined with sulfonylurea, shenqi jiangtang granules combined with metformin and jinlida granules combined with insulin had significant effects on reductions in FBG, 2hPG and clinical efficacy compared with western medicines alone, which included fasting blood glucose [MD=-2.17, 95%CI=(-2.50, -1.85)], blood sugar at 2 hours after a meal [MD=-1.94, 95%CI=(-2.23, -1.65)], and clinical curative effect [OR= 1.73, 95%CI=(0.59, 2.87)].

**Conclusions:**

TCM combined with CWM has a very significant effect on treating T2DM compared with CWM alone. According to the network meta-analysis, the best intervention measures of different TCMs for different outcome indicators were obtained.

**Systematic review registration:**

identifier 42022350372.

## 1 Introduction

Diabetes is a complex metabolic disease. The prevalence of diabetes has been increasing in recent years, and it has become the ninth leading cause of death throughout the world. This disease is a major public health problem affecting the entire world. Long-term exposure to a hyperglycemic environment in patients with diabetes can lead to abnormalities in various immune cells, thus negatively affecting multiple organs and leading to many common complications (1). The improper management of diabetes metabolism can also lead to serious long-term complications, including retinopathy, kidney disease, neuropathy, and cardiovascular disease, thus leading to significantly increased mortality. In 2019, the International Diabetes Federation reported that the number of people with diabetes is estimated at 463 million and will increase to 700 million by 2045 (2). Many risk factors and biological processes have been shown to contribute to the development of type 2 diabetes(T2DM), including hyperglycemia, hyperinsulinemia, insulin resistance, lipid accumulation, inflammation, oxidative stress, and adipokines (3). In addition, T2DM is caused by insulin resistance (IR), which leads to an increased demand for insulin in peripheral tissues and resulting in β-cell function failure. The core of the disease course is the impaired function of islet β-cells. However, there is a question as to how to improve the impaired function of islet β-cells and avoid the risk factors for T2DM.

The use of antidiabetic drugs is an ideal treatment method in clinical practice. Nevertheless, many drugs may lead to hypoglycemia, body fluid retention, cardiovascular disease, and other adverse reactions. However, there is a question as to how to choose the most effective treatment method exhibiting the most minor negative reactions. Currently, commonly used drugs for the treatment of diabetes include biguanides (metformin), sulfonylureas (SHD), dipeptidyl peptidase 4 inhibitors (DPP-IV), α-glucosidase inhibitors (AGI), and insulin (RI), among other drugs. According to the clinical efficacy, these drugs have very significant hypoglycemic effects. Some drugs, such as glucagon-like peptide-1 receptor agonist (GLP-1), can also reduce cardiovascular death and stroke in clinical diabetic patients, thus showing a perfect clinical effect. Drugs such as empagliflozin or liraglutide also reduce all-cause and cardiovascular mortality (4). With the development of traditional Chinese medicine (TCM), the clinical application of TCM combined with conventional western medicine (CWM) in treating T2DM is increasingly extensive. For example, studies have found that TCM can regulate the intestinal flora and improve glucose metabolism in patients with T2DM (5); moreover, the intestinal flora is essential for maintaining host health, and an increasing number of studies have been conducted on the treatment of diseases by targeting the intestinal flora with TCM, which fully demonstrates the advantages of TCM in this scenario. As characterized by syndrome differentiation and with a focus on symptom improvement, TCM has apparent benefits in the treatment of T2DM and its complications. TCM is characterized by multiple links, approaches, and targets in the prevention and treatment of diabetes, which is the advantage of TCM syndrome differentiation (6). TCM combined with CWM can reduce the risk of adverse reactions caused by a single treatment, such as gastrointestinal and hypoglycemia reactions. This study conducted a network meta-analysis on the intervention of TCM combined with CWM in T2DM, to explore the efficacy and safety ranking of the current treatment of T2DM, for guiding the optimal clinical treatment measures.

The intervention measures that were included in this study included shenqi jiangtang granules (SQJT), jinqi jiangtang tablets (JQJT), jinlida granules (JLD), tangmaikang granules (TMK), tianmai xiaoke tablets (TMXKP), tianqi jiangtang capsules (TQJT) and CWM intervention. The included TCM is recommended according to China’s Guidelines for the Prevention and Treatment of Type 2 Diabetes (2020 Edition). The selection of TCM has been unanimously agreed upon by clinical experts, and it is the medicine that possesses a significant effect on the treatment of T2DM. Due to the fact that it was approved by the State Food and Drug Administration in 1995, SQJT has successively entered the National Essential Medicine list and the National Medical Insurance List, and it has also won the National Torch project. A considerable amount of research data has been accumulated in the past 20 years. JQJT was approved by the Ministry of Health of the People’s Republic of China on August 19, 1992, and it won the third prize in science and technology progress in 1993. Moreover, JLD, which was launched in 2019, is an innovative TCM developed under the guidance of collateral disease theory and has a significant clinical market. The TCM included in this study has a mature basis for early clinical application, and has good effects on the treatment of T2DM. The specific ingredients of TCM are shown in **Table 1**. Traditional Western drugs included biguanides (metformin), sulfonylureas (SHD), dipeptidyl peptidase 4 inhibitors (DPP-IV), α-glucosidase inhibitors (AGI), insulin (RI), and glucagon-like peptide-1 receptor agonists (GLP-1). This study aimed to conduct a paired meta-analysis of head-to-head randomized controlled trials (RCTs) that were included in significant databases, followed by a network meta-analysis based on direct and indirect comparisons of standard controls (conventional western medicines). Currently, the indirect treatment of different TCMs combined with CWM is relatively small in clinical practice. In this study, a network meta-analysis was used to conduct direct and indirect analyses of the intervention of different TCMs combined with CWM to explore the optimal therapeutic means for the clinical treatment of T2DM.

**Table 1:** Incorporate traditional Chinese medicine components.

Name	Composition
Shenqi Jiangtang Granules	Ginsenoside, Schisandra chinensis (Turcz.) Baill., Hedysarum Multijugum Maxim., Rhizoma Dioscoreae, Rehmannia glutinosa (Gaetn.) Libosch. ex Fisch. et Mey., Rubus idaeus L., Poria Cocos(Schw.) Wolf., Ophiopogon japonicus (Linn. f.) Ker-Gawl., Trichosanthis Radix, Alisma Orientale (Sam.) Juz., Lycii Fructus
Jin Qi Jiangtang Tablets	Coptidis Rhizoma, Hedysarum Multijugum Maxim., Lonicera japonica Thunb.
Jinlida granules	Panax Ginseng C. A. Mey., Polygonati Rhizoma, Atractylodes Lancea (Thunb.)Dc., Sophora flavescens, Ophiopogon japonicus (Linn. f.) Ker-Gawl., Rehmannia glutinosa (Gaetn.) Libosch. ex Fisch. et Mey., Fallopia multiflora (Thunb.) Harald., Cornus Officinalis Sieb. Et Zucc., Poria Cocos(Schw.) Wolf., Eupatorium fortunei Turcz., Coptidis Rhizoma, Anemarrhenae Rhizoma, Epimedii Folium, Radix Salviae, Radix Puerariae, Litchi chinensis Sonn., Cortex Lycii
Tangmaikang granules	Hedysarum Multijugum Maxim., Rehmannia glutinosa (Gaetn.) Libosch. ex Fisch. et Mey., Radix Paeoniae Rubra, Radix Puerariae, Morus alba L., Epimedii Folium
Tianmai Xiaoke Tablets	Schisandra chinensis (Turcz.) Baill., Ophiopogon japonicus (Linn. f.) Ker-Gawl., Trichosanthis Radix, Chromium picorinate
Tianqi Jiangtang Capsule	Hedysarum Multijugum Maxim., Trichosanthis Radix, Ligustri Lucidi Fructus, Dendrobium nobile Lindl., Panax Ginseng C. A. Mey., Cortex Lycii, Coptidis Rhizoma, Cornus Officinalis Sieb. Et Zucc., Ecliptae Herba, Galla Chinensis

## 2 Methods

### 2.1 Literature search

This network meta-analysis was conducted according to the PRISMA Extended Statement, and it was performed step-by-step in strict accordance with the PRISMA Checklist. This study has been registered on the International Prospective Register for Systems Evaluation (PROSPERO) website as CRD number 42022350372.

The literature search time of this study ranged from January 2010 to October 2022. The literature search was conducted by searching the CNKI, WanFang, VIP, CBM, PubMed, Embase, and Web of Science databases and was screened according to the inclusion and exclusion criteria. The primary screening direction was a randomized controlled trial (RCT) of TCM combined with CWM in the treatment of T2DM. The included TCMs included SQJT, JQJT, JLD, TMK, TQJT, and TMXKP. In addition, the included CWMs were as follows: sulfonylureas (SHD), biguanides (metformin), dipeptidyl peptidase-4, α-glucosidase inhibitors (DPP-IV), alpha-glucosidase inhibitors (AGI), insulin (RI), and glucagon-like peptide-1 (GLP-1),which were uniformly divided into the category of CWM. Two researchers independently conducted a literature search, and the Chinese database used “Shenqi Jiangtang Granules”, “Jinqi Jiangtang tablets”, “Jinlida granules”, “Tangmaikang granules”, “Tianqi Jiangtang capsules”, “Tianmai Xiaoke tablets”, “Conventional Western medicine”, “Type 2 diabetes mellitus”, for the literature search. English databases under “shenqi jiangtang”, “jinqi jiangtang”, “jinlida”, “tangmaikang”, “tianqi jiangtang”, “tianmai xiaoke”, and “diabetes”, were retrieved in the form of subject words + free words. Two researchers independently conducted a literature search. After selecting the weight, some types of literature were screened according to the title and abstract. Finally, the final selection was performed according to the reading of the full text to obtain the final included literature.

### 2.2 Criteria for inclusion

The included research criteria were as follows. (1) Research on the treatment of T2DM with TCM combined with CWM that met the requirements of this paper, with traditional Chinese medicine including SQJT, JQJT, JLD, TMK, TQJT, and TMXKP and conventional Western medicine including SHD, metformin, DPP-IV, AGI, RI, and GLP-1. (2) Randomized controlled clinical trials (RCTs). (3) The intervention time being at least 2 months. (4) The included literature including at least one outcome indicator required by this study. (5) Adults with T2DM (≥18 years old). (6) Research data in the literature that was able to be obtained through public platforms.

### 2.3 Exclusion criteria

The exclusion criteria for this study were as follows: (1) the publication was an animal experiment, an academic conference, a paper, or a nonrandomized controlled trial (RCT). (2) TCM and CWM that did not meet the requirements of the study. (3) literature published as an abstract only. (4) a sole focus on laboratory findings that were not RCTs, reviews, or reports. (5) repeated publications of literature. (6) the literature with complete data that could not be obtained after contacting the author.

### 2.4 Data extraction and quality assessment

Two researchers independently extracted the data of the included literature and assessed the bias risk of the included literature. Data extraction was performed according to the Excel table, which was prepared in advance. The extraction contents of the table was comprised of the author of the included literature, publication year, intervention group, age, sex, course of the disease, the total number of included studies, the grouping method of the clinical studies, whether the blind method was implemented, outcome indicators, intervention and follow-up times, specific measures of intervention, and the value of the outcome indicators, among other contents. If there was a dispute, a third researcher discussed it with the other researchers. We conducted a risk assessment of the included studies by using the Cochrane ROB Bias risk assessment tool that was used to assess the RCTs. The risk levels were high, medium, and low risk, and the literature was independently evaluated by two investigators by using the same standardized methodology. The risk of bias in the included literature was evaluated by considering randomization, assignment concealment, whether participants and researchers were blinded, the blind evaluation of outcome indicators, and selective reporting.

### 2.5 Outcomes

According to the consensus of clinical experts and previous experience, we selected FBG, 2hPG, HbA1c, clinical efficacy, and incidence of adverse reactions as important outcome indicators to evaluate the effectiveness of TCM combined with CWM in the intervention of T2DM.

### 2.6 Statistical analysis

In this study, a combination of traditional meta-analysis and network meta-analysis was used to analyze the included literature. Traditional meta-analysis aims to explore the efficacy of direct interventions of different TCMs combined with CWM in the treatment of T2DM. In contrast, network meta-analysis is an overall comparison of various intervention measures; specifically, direct and indirect comparisons. We combined RevMan 5.4 software and Stata15 software package “net meta” to analyze the literature, wherein we used the I^2^ index to evaluate the heterogeneity among the studies and hypothesized homogeneity. For binary variables, the OR index was used. For continuous variables, the MD index was used. Moreover, we used 95% confidence intervals (CIs) to determine the clinical effect of the included study. We performed a frequency network meta-analysis by using Stata software to analyze indirect comparisons between different interventions. The probability of an intervention being the safest and most effective was estimated by using the area under cumulative ranking, and the relative ranking probability of different interventions was displayed. The effect values and 95%CIs of pair-to-pair comparisons between the interventions were clearly expressed by using league tables, and the inconsistencies of the results were confirmed using the node-splitting method and Bayes P value. However, inconsistencies could not be assessed because the network meta-analysis did not close the loop. Furthermore, a sensitivity analysis was used to determine the risk of bias in the included studies. According to the differences among the literature included in the intensive reading, the similarity hypothesis was formulated. *Via* the various statistical analyses, the different effects of intervention measures and clinical efficacy were completely analyzed.

## 3 Results

### 3.1 The process of literature retrieval and the primary research characteristics of the included literature

**Figure 1:**
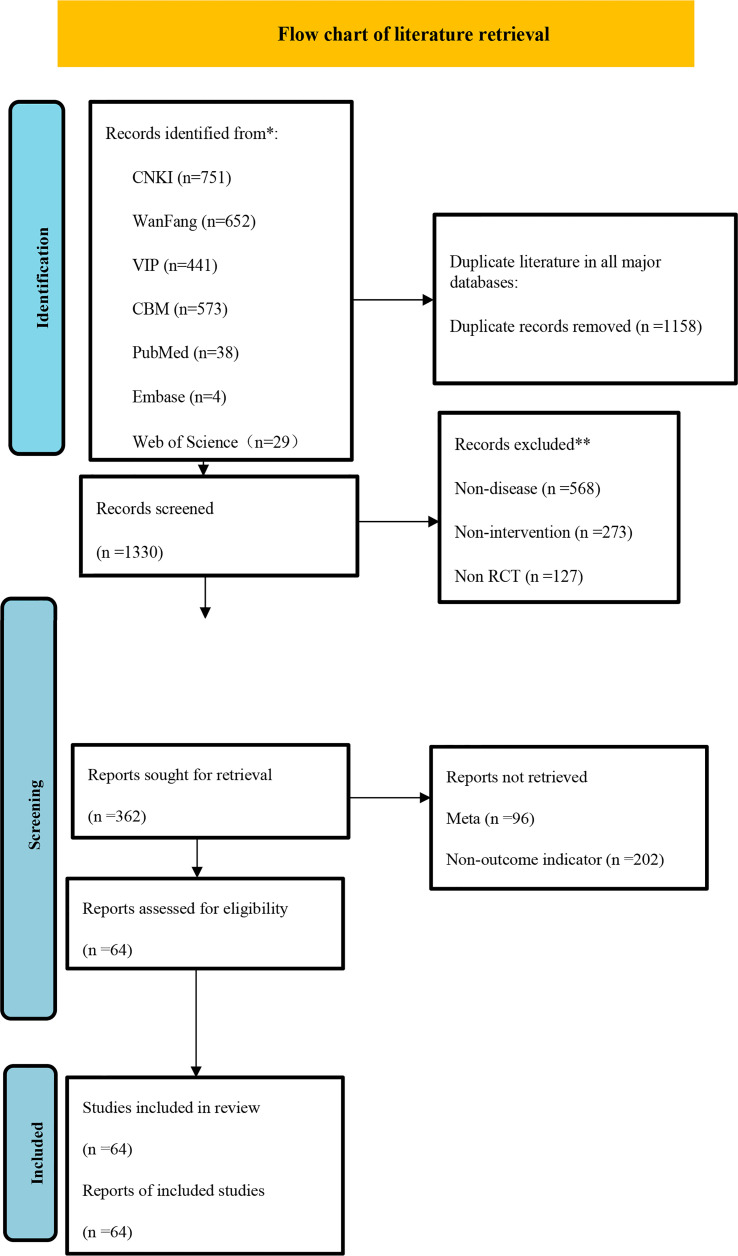
Flow chart of literature retrieval.

After literature retrieval in various major databases according to strict exclusion criteria, 64 studies were finally included. Two researchers independently extracted the included literature according to Excel tables. The flow charts and essential characteristics of the included literature are shown in **Figure 1** and **Table 2** respectively.

**Table 2:** Basic features included in the study.

Study	Year	Age	Sample size	Duration of intervention	Interventions	Outcome
Wei Shao (7)	2022	58.26 ± 1.37vs55.53 ± 1.63	96	3 months	C: AGI/E: SQJT+AGI	①②③④⑤
Zhizhong Wang (8)	2013	59.2 ± 6.5vs57.1 ± 5.5	60	2 months	C: AGI/E: SQJT+AGI	①②③④
Yingfang Liu (9)	2016	63.1 ± 7.6vs62.8 ± 7.4	116	1 month	C: AGI/E: SQJT+AGI	①③④⑤
Quanbing Xia (10)	2016	72.98 ± 5.61vs73.62 ± 6.54	120	4 months	C: AGI/E: SQJT+AGI	①②③④⑤
Linghua Guan (11)	2018	61.2 ± 11.4vs60.5 ± 10.7	90	2 months	C: SHD/E: SQJT+SHD	①②④⑤
Yuan Yuan (12)	2017	65.6 ± 7.7vs67.8 ± 8.7	64	6 months	C: SHD/E: SQJT+SHD	①②③⑤
Senyue Zhang (13)	2018	66.1 ± 6.3vs65.8 ± 6.4	196	2 months	C: SHD/E: SQJT+SHD	①②
Shuhui Guo (14)	2016	61.23 ± 7.32vs64.18 ± 7.73	110	2 months	C: SHD/E: SQJT+SHD	①②③④⑤
Wei Zhang (15)	2017	64.15 ± 3.25vs64.32 ± 3.32	96	2 months	C: SHD/E: SQJT+SHD	①②③④
Feilong gao (16)	2020	62.43 ± 5.02vs62.37 ± 4.97	86	3 months	C: DPP-IV/E: SQJT+DPP-IV	①②
Yanju Liu (17)	2018	50.97 ± 6.49vs51.90 ± 5.98	120	6 months	C: DPP-IV/E: SQJT+DPP-IV	①②⑤
Qiwei Ren (18)	2019	56.32 ± 3.29	92	3 months	C: DPP-IV/E: SQJT+DPP-IV	①②③
Huiping Liu (19)	2017	56.55 ± 7.17vs55.30 ± 7.36	40	3 months	C: Metformin/E: SQJT+ Metformin	①③④
Changjun Cai (20)	2014	50.3 ± 5.3vs51.3 ± 5.1	76	3 months	C: Metformin/E: SQJT+ Metformin	①②③
Yijie Wang (21)	2016	56.4 ± 5.2vs58.2 ± 4.1	64	3 months	C: Metformin/E: SQJT+ Metformin	①②③④
Shengyong Liu (22)	2020	64.73 ± 7.56vs64.73 ± 7.56	101	1 month	C: Metformin/E: SQJT+ Metformin	①②③④
Huajun Yang (23)	2019	53.02 ± 8.47vs51.28 ± 7.63	92	3 months	C: Metformin/E: SQJT+ Metformin	①②③
Mengyao Liu (24)	2017	43.23 ± 1.24vs42.42 ± 1.12	80	3 months	C: Metformin/E: SQJT+ Metformin	①②③
Jinrui Sun (25)	2020	62.3 ± 2.81vs61.2 ± 7.20	74	3 months	C: Metformin/E: SQJT+ Metformin	①②③④
Fengling Sui (26)	2019	54.31 ± 5.11vs53.25 ± 4.7	104	3 months	C: Metformin/E: SQJT+ Metformin	①②③④
Shiyu Chen (27)	2019	55.2 ± 3.5vs54.3 ± 3.7	104	3 months	C: Metformin/E: SQJT+ Metformin	①②③⑤
Yingli Fan (28)	2014	/	80	3 months	C: Metformin/E: SQJT+ Metformin	①②③④
Guanghai Liu (29)	2018	67.2 ± 9.4vs66.8 ± 10.2	60	3 months	C: Metformin/E: SQJT+ Metformin	①②③
Lei Zhang (30)	2019	57.13 ± 9.86vs57.46 ± 9.74	120	3 months	C: Metformin/E: SQJT+ Metformin	①②③④
Shuijiao Li (31)	2022	57.49 ± 4.35	94	3 months	C: RI/E: SQJT+RI	①②④
Ying Du (32)	2022	50.69 ± 8.89vs49.69 ± 8.21	142	3 months	C: RI/E: SQJT+RI	①②③
Xiaolin Li (33)	2021	/	104	3 months	C: RI/E: SQJT+RI	①③④
Ge Lv (34)	2017	51.66 ± 1.97vs51.30 ± 1.33	86	3 months	C: RI/E: SQJT+RI	①②③④
Mingke Gao (35)	2021	54.9 ± 10.3vs56.3 ± 11.1	112	3 months	C: RI/E: JQJT+RI	①③
Tao Li (36)	2018	38.17 ± 2.27vs37.24 ± 3.05	96	2 months	C: RI/E: JQJT+RI	①②③④
Tao Li2 (37)	2012	46.5 ± 6.3vs43.8 ± 4.7	100	3 months	C: RI/E: JQJT+RI	①②③
Qingchun Yao (38)	2014	54.5 ± 6.9vs55.5 ± 6.1	68	2 months	C: RI/E: JQJT+RI	①②③④
Xiaoming Li (39)	2017	40.4 ± 3.1vs39.3 ± 3.8	86	2 months	C: RI/E: JQJT+RI	①②③
Xibo Hu (40)	2022	66.5 ± 6.8vs66.8 ± 6.2	90	3 months	C: DPP-IV/E: JLD+DPP-IV	①②③④⑤
Jing Wu (41)	2021	54.36 ± 8.74vs54.71 ± 8.39	94	3 months	C: DPP-IV/E: JLD+DPP-IV	①②③④⑤
Yange Tang2 (42)	2017	57.4 ± 2.0vs57.3 ± 2.2	110	2 months	C: DPP-IV/E: JLD+DPP-IV	①②
Xuehong Zhou (43)	2020	55.74 ± 1.53vs55.12 ± 1.07	92	2 months	C: GLP-1/E: JLD+GLP-1	①②③④
Suzhen Zhang (44)	2018	53.64 ± 1.35vs53.57 ± 1.28	90	3 months	C: GLP-1/E: JLD+GLP-1	①②③④
Lin Zhou (45)	2020	32.48 ± 2.28vs32.51 ± 2.25	78	3 months	C: Metformin/E: JLD+ Metformin	①②④
Hanquan Li (46)	2017	72.96 ± 2.87vs72.16 ± 2.14	92	3 months	C: Metformin/E: JLD+ Metformin	①③④
Yange Tang (47)	2017	51.83 ± 12.61vs51.11 ± 11.4	70	2 months	C: Metformin/E: JLD+ Metformin	①②③
Aihua Wu (48)	2019	63.56 ± 10.11vs62.56 ± 10.2	60	6 months	C: Metformin/E: JLD+ Metformin	①②③⑤
Xiyue Xing (49)	2021	57.58 ± 2.13vs57.70 ± 2.24	100	3 months	C: Metformin/E: JLD+ Metformin	①②③④
Guanzhen Wang (50)	2018	44.68 ± 5.79vs43.7 ± 5.86	106	2 months	C: Metformin/E: JLD+ Metformin	①②③⑤
Yanfen Fu (51)	2019	45.09 ± 3.52vs44.12 ± 4.31	193	3 months	C: Metformin/E: JLD+ Metformin	①②③④⑤
Quanmin Ma (52)	2014	79.3 ± 3.4vs80.3 ± 4.8	80	3 months	C: Metformin/E: JLD+ Metformin	①②③④
Wei Xiao (53)	2019	68.62 ± 10.25vs69.65 ± 10.8	82	3 months	C: SHD/E: JLD+SHD	①②③
Hong Liu (54)	2018	45.94 ± 9.86vs46.73 ± 7.18	120	2 months	C: SHD/E: JLD+SHD	①②③④
Liangqi Luo (55)	2018	45.3 ± 3.2vs46.1 ± 3.3	60	3 months	C: SHD/E: JLD+SHD	①②③④
Wei Zhu (56)	2021	58.70 ± 1.25vs58.59 ± 1.31	120	2 months	C: RI/E: JLD+RI	①②③⑤
Junchen Huang (57)	2020	51.04 ± 8.31vs54.49 ± 6.15	98	3 months	C: RI/E: JLD+RI	①②③④⑤
Xiaoyan Luo (58)	2018	54.7 ± 5.1vs53.9 ± 5.4	91	2 months	C: RI/E: JLD+RI	①②③④
Yuan Lin (59)	2017	54.94 ± 6.31vs53.32 ± 6.75	130	2 months	C: Metformin/E: TMK+ Metformin	①②③
Manli Hu (60)	2015	54.3 ± 3.3vs55.1 ± 2.6	230	2 months	C: Metformin/E: TMK+ Metformin	①②③
Hui Li (61)	2019	52.34 ± 3.58vs51.08 ± 4.12	80	6 months	C: Metformin/E: TMK+ Metformin	①②③⑤
Wei Du (62)	2010	44.25 ± 1.65	142	3 months	C: Metformin/E: TMK+ Metformin	①②③
Dajun Lou (63)	2016	56.3 ± 6.7	120	3 months	C: Metformin/E: TMXKP+ Metformin	①②③⑤
Yan Shao (64)	2017	/	80	3 months	C: Metformin/E: TMXKP+ Metformin	①②③
Fengli Huang (65)	2016	45.6 ± 9.8	86	3 months	C: Metformin/E: TMXKP+ Metformin	①②③⑤
Wanrong Yu (66)	2019	/	60	3 months	C: Metformin/E: TMXKP+ Metformin	①②③
Ruijun Qin (67)	2016	72.28 ± 6.49vs72.11 ± 6.46	82	3 months	C: Metformin/E: TMXKP+ Metformin	①②
Yao Li (68)	2011	55.3 ± 6.98vs56.46 ± 9	80	3 months	C: Metformin/E: TQJT+ Metformin	①②③
Yong Cao (69)	2015	50.08 ± 7.21vs49.69 ± 7.12	80	2 months	C: Metformin/E: TQJT+ Metformin	①③
Fengmei Lian (70)	2011	/	79	3 months	C: Metformin/E: TQJT+ Metformin	①②③

①FBG ②2hPG ③HbA1c ④Clinical efficacy ⑤Incidence of adverse reactions.

“/” indicates that the intervention group is distinguished from the control group.

### 3.2 Risk of bias in included studies

**Figure 2:**
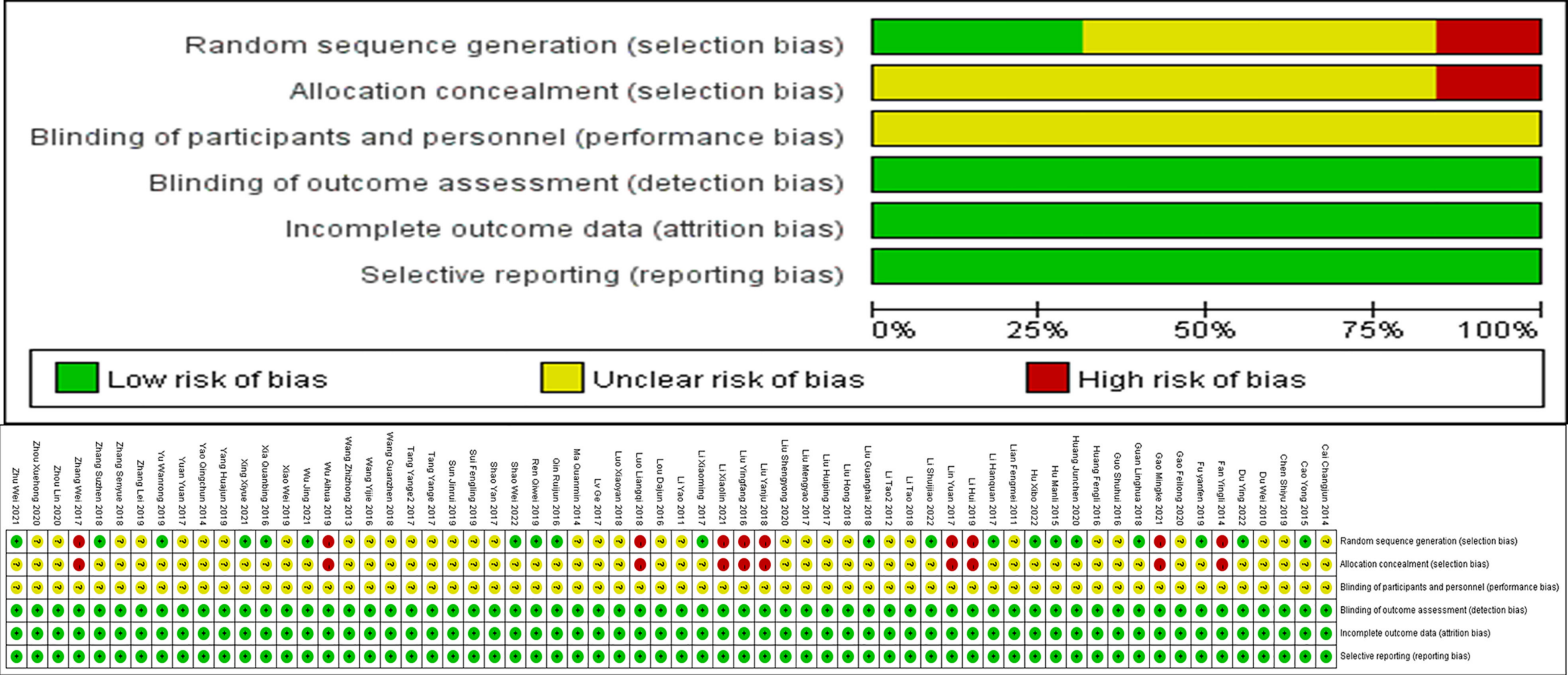
Risk of bias in included studies.

This study conducted a detailed assessment of the risk of bias using Cochrane assessment methods. Random sequence generation, Allocation concealment, Blinding of participants and personnel, Blinding of outcome assessment, Incomplete outcome data, and Selective reporting were analyzed, and the results are shown in **Figure 2**.

### 3.3 Traditional and network meta-analyses of outcome measures

#### 3.3.1 FBG

##### 3.3.1.1 Traditional meta-analysis and network meta-analysis and league table

**Figure 3:**
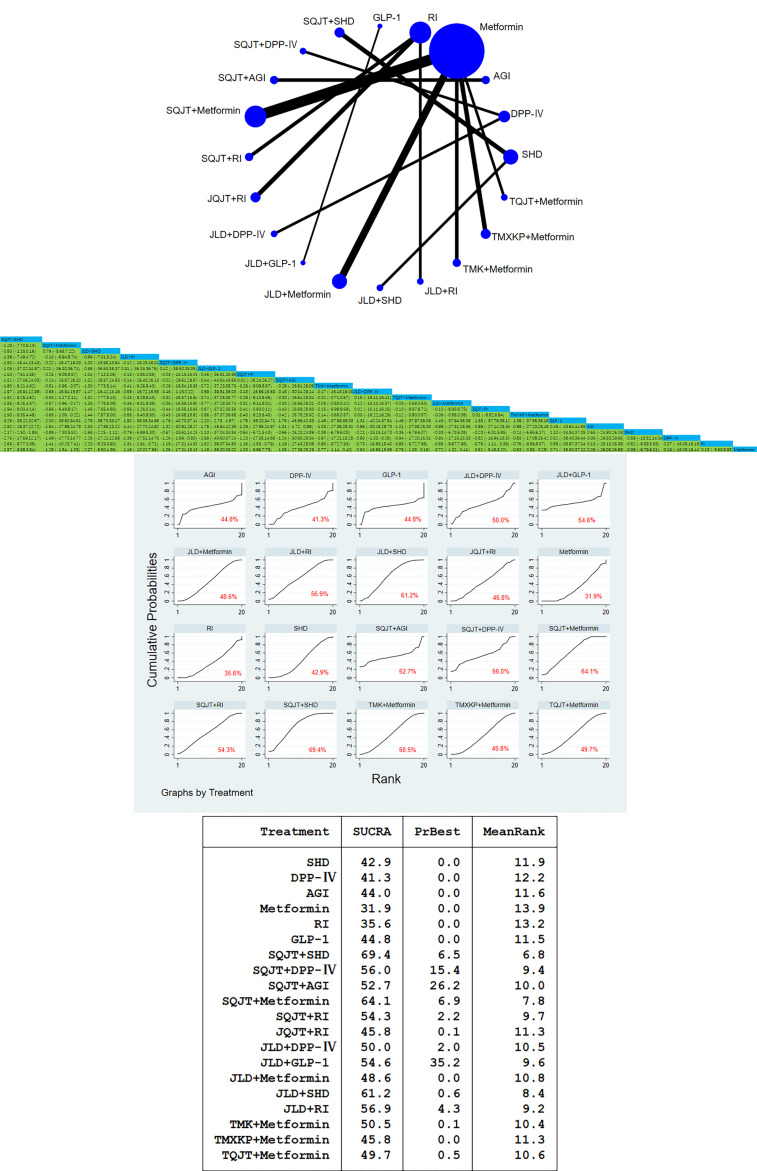
FBG network diagram, league diagram, forest diagram, SUCRA diagram.

64 studies, a total of 6204 patients, all included the outcome index FBG. After traditional meta-analysis and network meta-analysis of FBG, it was concluded that the number of studies on SQJT combined with Metformin and JLD combined with Metformin was the largest. According to the result of direct and indirect comparison of TCM combined with CWM treatment: TCM combined with different CWM has significant hypoglycemic effect compared with simple CWM, among which, SQJT combined with SHD and JLD combined with SHD compared with simple SHD have hypoglycemic effect value [MD=-2.17, 95%CI=(-2.50, -1.85)], [MD=-1.68,95%CI=(-2.25,-1.11)]; SQJT combined with Metformin are better than Metformin alone, TMXKP combined with Metformin and TMK combined with Metformin, hypoglycemic effect value of respectively [MD=- 1.28, 95%CI=(-1.54, -1.03)], [MD=-0.65, 95%CI=(-1.09, -0.22)], [MD=-0.51, 95%CI=(-0.96, -0.07)]. The hypoglycemic effect of JLD combined with RI, SQJT combined with RI and JQJT combined with RI was significantly better than that of RI alone, effect value of respectively [MD=- 1.31, 95%CI=(-1.91, -0.71)], [MD=-1.16, 95%CI=(-1.53, -0.79)], [MD=-0.75, 95%CI=(-1.11, -0.38)] (**Figure 3**).

##### 3.3.1.2 Cumulative curves under the area chart sort

According to the area ranking diagram under the FBG accumulation curve of the outcome index, SQJT combined with SHD had the best effect in reducing the FBG content among the intervention measures, while the hypoglycemic effect of CWM alone was significantly lower than that of other TCM combined with CWM. The sequence of hypoglycemic effect of comprehensive intervention measures is as follows: SQJT+SHD > SQJT+Metformin > JLD+SHD > JLD+RI > SQJT+DPP-IV > JLD+GLP-1 > SQJT+RI > SQJT+AGI > TMK+Metformin > JLD+DPP-IV > TQJT+Metformin > JLD+Metformin > JQJT+RI > TMXKP+Metformin > GLP-1 > AGI > SHD > DPP-IV > RI > Metformin, which proves that TCM combined with CWM has a significant effect on reducing FBG content, and SQJT combined with SHD has the most significant effect (**Figure 3**).

#### 3.3.2 2hPG

##### 3.3.2.1 Traditional meta-analysis and network meta-analysis and league table

**Figure 4:**
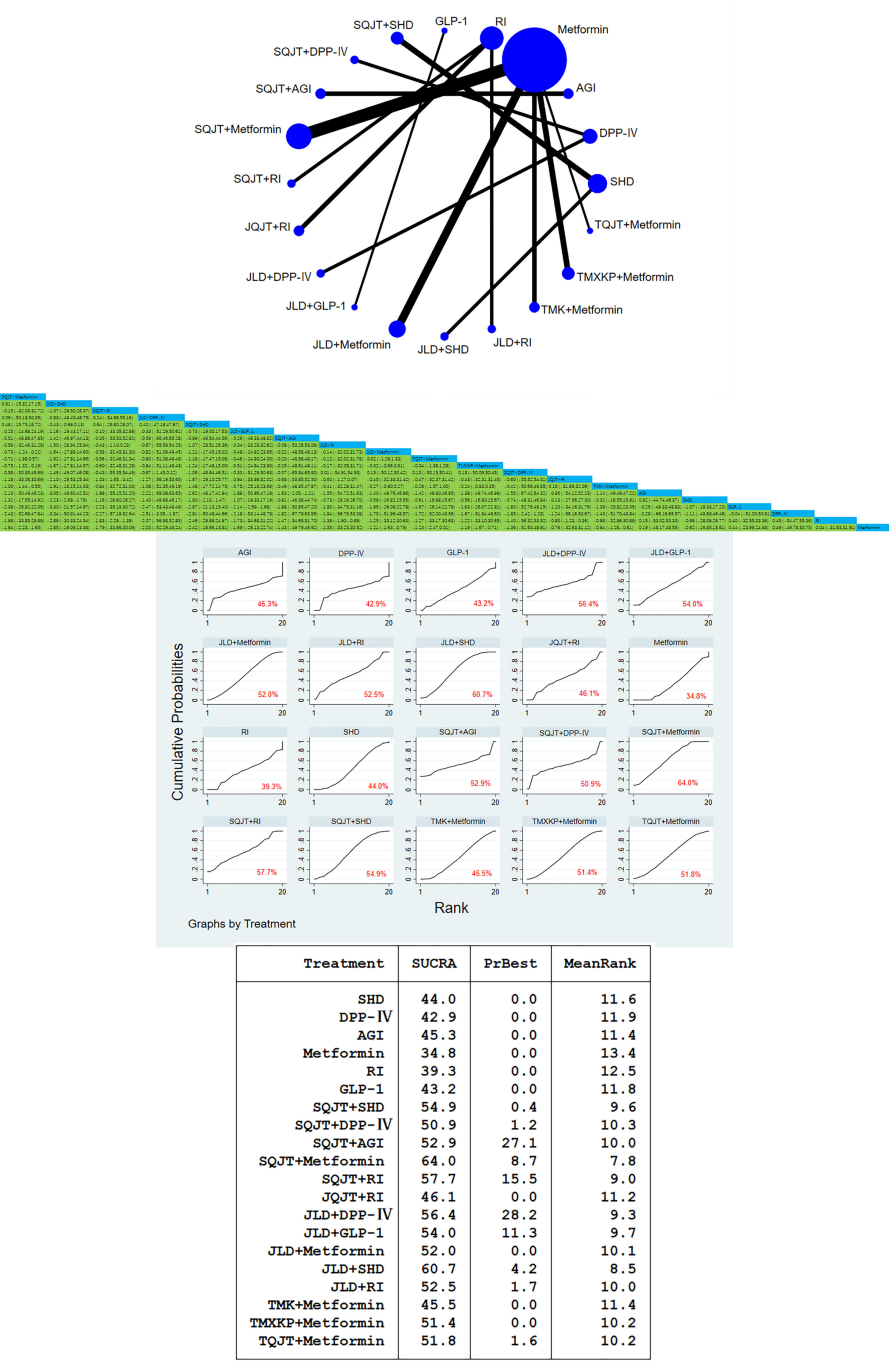
2hPG network diagram, league diagram, forest diagram, SUCRA diagram.

A total of 59 studies, involving a total of 5772 patients, included 2-hour postprandial glucose outcomes. In this study, traditional meta-analysis and network meta-analysis of blood glucose 2 hours after meals showed that there were most studies on the comparison between SQJT combined with Metformin, JLD combined with Metformin and Metformin alone, and most of the studies were direct comparison between TCM combined with CWM and CWM, while there was no direct comparison between different TCM. According to the result of 2hPG, SQJT combined with Metformin, JLD combined with Metformin, TMXKP combined with Metformin and TMK combined with Metformin have significant hypoglycemic effects compared with Metformin alone, effect value of respectively [MD=-1.94, 95%CI=(-2.23, -1.65)], [MD=-1.21, 95%CI=(-1.63, -0.79)], [MD=-1.19, 95%CI=(-1.67, -0.71)], [MD=-0.94, 95%CI=(-1.28,-0.61)]; The therapeutic effect of JLD combined with SHD and SQJT combined with SHD was significantly better than that of SHD alone, and the effect values were [MD=-2.23,95%CI=(-2.69,-1.78)] and [MD=-1.80,95%CI=(-2.13,-1.47)]; The hypoglycemic effect of SQJT combined with RI and JLD combined with RI was better than that of RI alone, and the effect values were [MD=-1.83,95%CI=(-2.26,-1.39)] and [MD=-1.39,95%CI=(-1.90,-0.89), respectively]; The above are the direct comparison between different TCM combined with CWM and simple CWM treatment, while the indirect comparison results between different TCM combined with CWM show that, the hypoglycemic effect of SQJT combined with Metformin is better than that of JLD combined with Metformin, TMXKP combined with Metformin and TMK combined with Metformin, effect value of respectively [MD=-0.73, 95%CI=(-1.24, -0.22)], [MD=-0.75, 95%CI=(-1.32, -0.19)], [MD=-1.00, 95%CI=(-1.44, -0.55)]; The therapeutic effect of SQJT combined with RI was better than that of JQJT combined with RI, and the effect value was [MD=-1.03,95%CI=(-1.65,-0.42)] (**Figure 4**).

##### 3.3.2.2 Cumulative curves under the area chart sort

According to the outcome index, the area sorting diagram under the 2hPG accumulation curve showed that SQJT combined with Metformin had the most significant effect on reducing the 2hPG. The therapeutic effect ranking of various interventions is as follows: SQJT+Metformin > JLD+SHD > SQJT+RI > JLD+DPP-IV > SQJT+SHD > JLD+GLP-1 > SQJT+AGI > JLD+RI > JLD+Metformin > TQJT+Metformin > TMXKP+Metformin > SQJT+DPP-IV > JQJT+RI > TMK+Metformin > AGI > SHD > GLP-1 > DPP-IV > RI > Metformin, which proves that TCM combined with CWM has a significant effect on reducing 2hPG compared with CWM alone. The league table clearly shows the 95%CI for each intervention (**Figure 4**).

#### 3.3.3 HbA1c

##### 3.3.3.1 Traditional meta-analysis and network meta-analysis and league table

**Figure 5:**
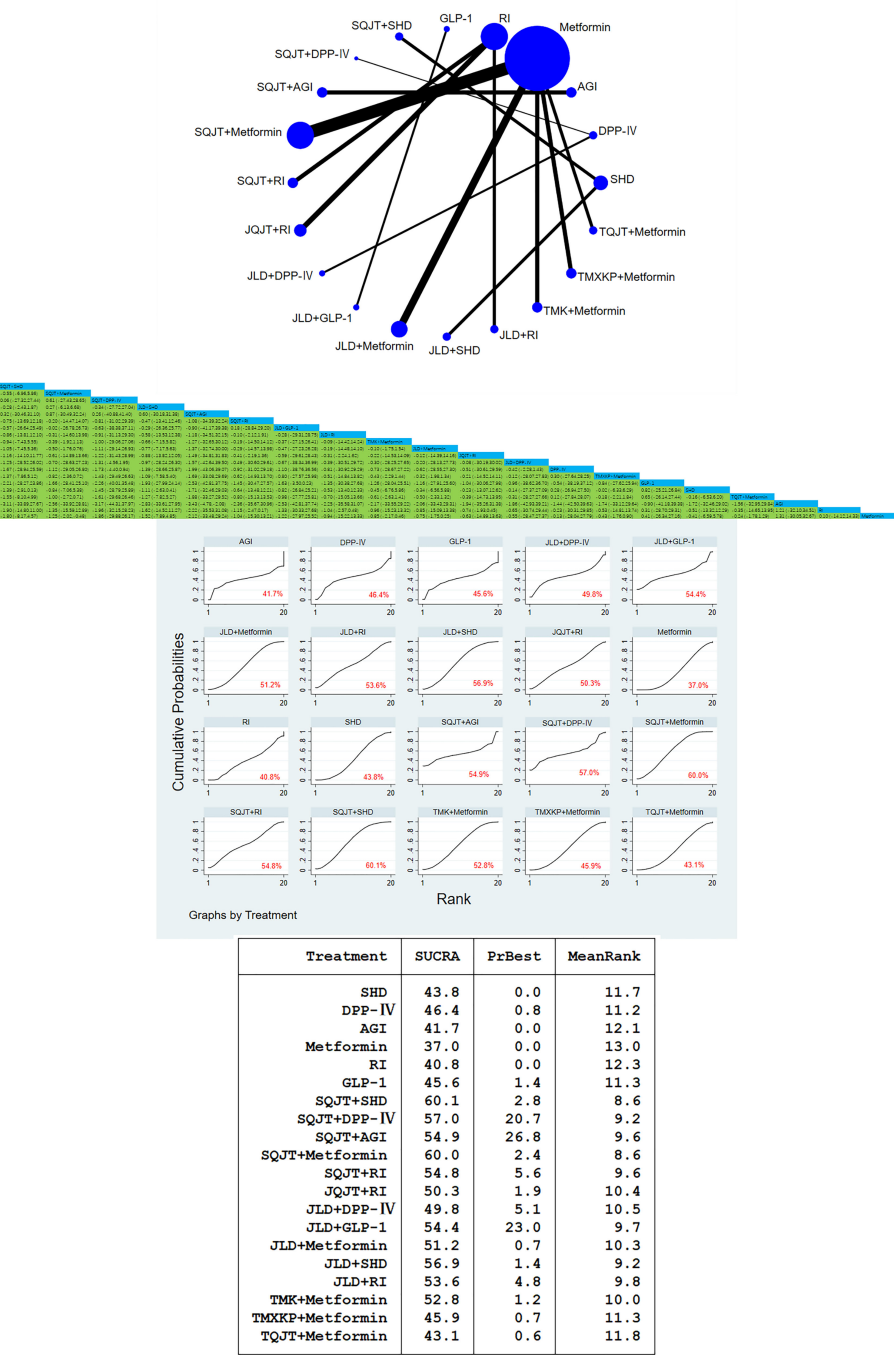
HbA1c network diagram, league diagram, forest diagram, SUCRA diagram.

Fifty-seven studies with a total of 5,432 patients included HbA1c outcome measures. In this study, traditional meta-analysis and network meta-analysis of HbA1c showed that: Among the direct comparative studies of TCM combined with CWM and CWM alone, SQJT combined with Metformin and JLD combined with Metformin were the most relevant studies. According to the result of meta-analysis, SQJT combined with Metformin had a significant effect on reducing the HbA1c content compared with Metformin alone, and the effect value was [MD=-1.25, 95%CI=(-2.02, -0.48)]; SQJT combined with AGI had significant effect compared with AGI alone, and the effect value was [MD=-3.43, 95%CI=(-4.78, -2.08)]. There was no significant difference in the comparison of other interventions (**Figure 5**).

##### 3.3.3.2 Cumulative curves under the area chart sort

According to the area ranking diagram under the cumulative curve of the glycosylated hemoglobin content of the outcome index, SQJT combined with SHD have the most significant effect on reducing the HbA1c, and the therapeutic effects of various intervention measures are ranked as follows: SQJT+SHD > SQJT+Metformin > SQJT+DPP-IV > JLD+SHD > SQJT+AGI > SQJT+RI > JLD+GLP-1 > JLD+RI > TMK+Metformin > JLD+Metformin > JQJT+RI > JLD+DPP-IV > DPP-IV > TMXKP+Metformin > GLP-1 > SHD > TQJT+Metformin > AGI > RI > Metformin, it was proved that TCM combined with CWM had a significant effect on reducing the level of HbA1c compared with CWM alone (**Figure 5**).

#### 3.3.4 Incidence of adverse reactions

##### 3.3.4.1 Traditional meta-analysis and network meta-analysis and league table

**Figure 6:**
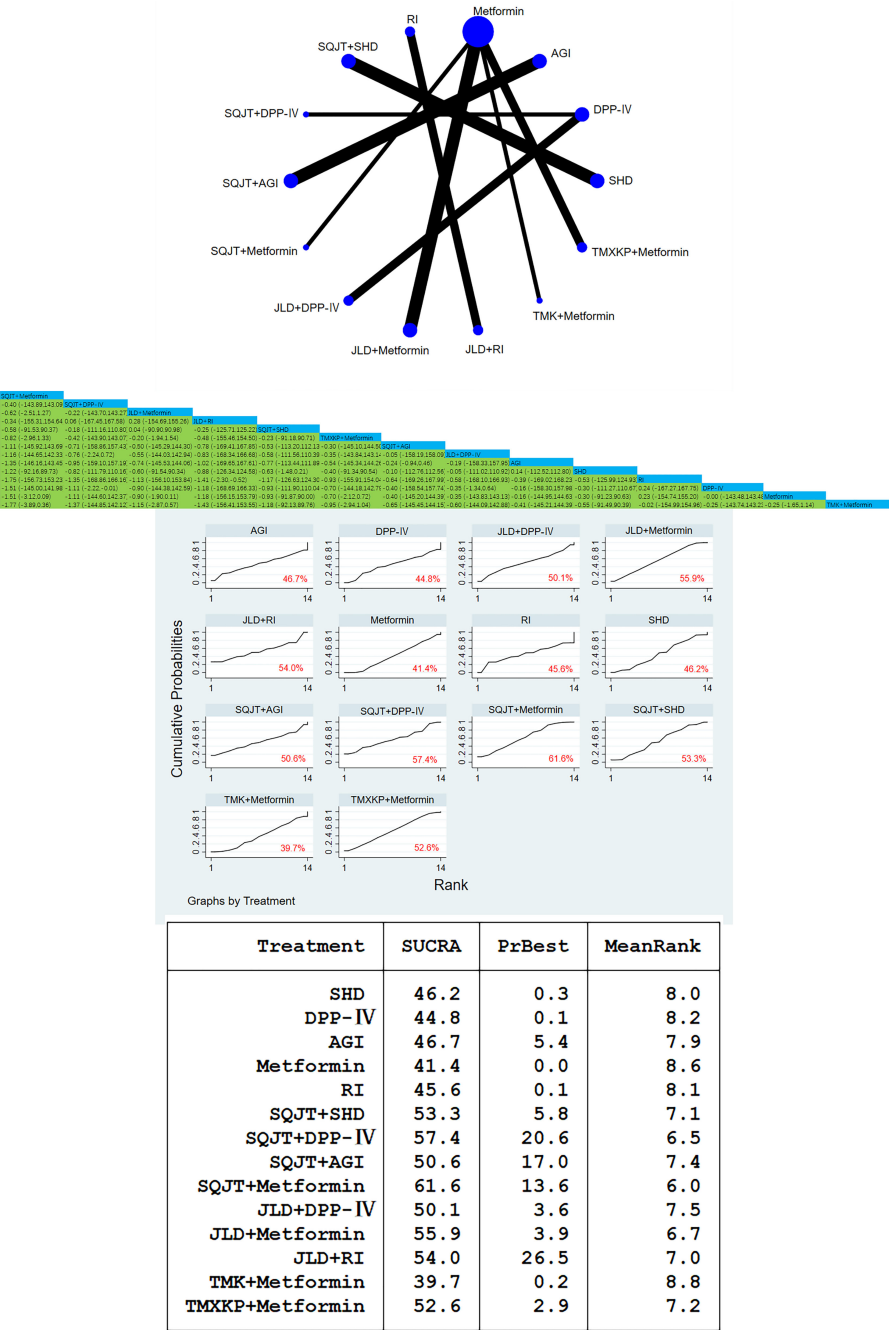
The incidence of adverse reactions was netted, league, forest and SUCRA.

Eighteen RCTs, involving a total of 1867 patients, included the incidence of adverse reactions as an outcome indicator. In this study, traditional meta-analysis and network meta-analysis were performed on the incidence of adverse reactions. According to the result, SQJT combined with DPP-IV had more safety and lower incidence of adverse reactions compared with CWM alone, and the effect value was [OR=-1.11, 95%CI=(-2.22, -0.01)]; Compared with RI alone, JLD combined with RI had better safety, and the effect value was [OR=-1.41, 95%CI=(-2.30, -0.52)], there was no significant difference between other interventions (**Figure 6**).

##### 3.3.4.2 Cumulative curves under the area chart sort

According to the area ranking graph under the cumulative curve of adverse reaction incidence of outcome indicators, JLD combined with Metformin was the best intervention measure, and the best ranking of various intervention measures was as follows: SQJT+Metformin > SQJT+DPP-IV > JLD+Metformin > JLD+RI > SQJT+SHD > TMXKP+Metformin > SQJT+AGI > JLD+DPP-IV > AGI > SHD > RI > DPP-IV > Metformin > TMK+Metformin (**Figure 6**).

#### 3.3.5 Clinical efficacy

##### 3.3.5.1 Traditional meta-analysis and network meta-analysis and league table

**Figure 7:**
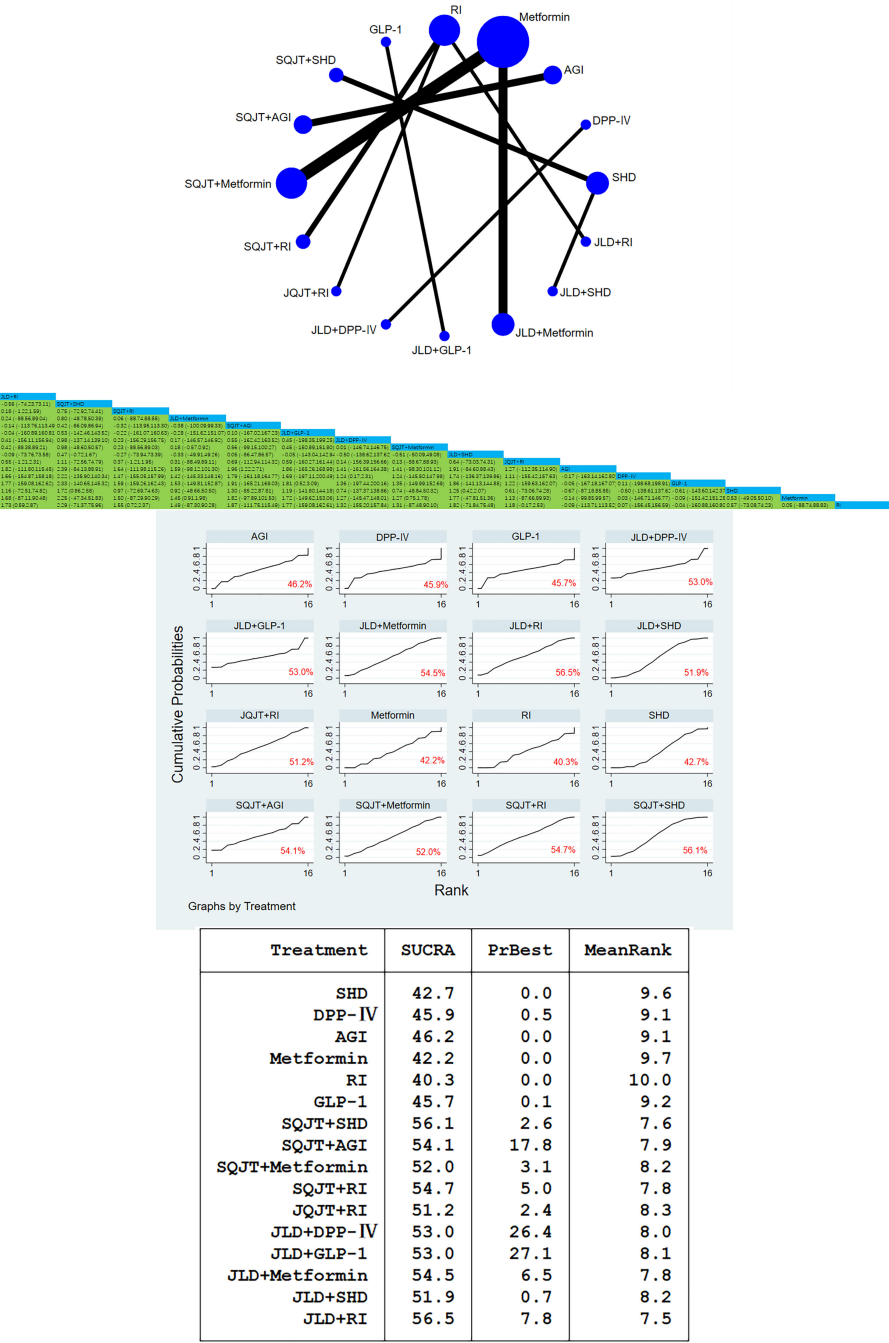
Clinical efficacy of network, league, forest, SUCRA chart.

Thirty-two RCTs, with a total of 3,045 patients, included outcome measures of clinical efficacy. In this study, traditional meta-analysis and network meta-analysis of clinical efficacy were conducted. According to the result, compared with RI alone, JLD combined with RI and SQJT combined with RI showed better clinical efficacy, and the effect values were [OR=1.73, 95%CI= (0.59, 2.87)] and [OR=1.55, 95%CI=(0.72, 2.37)]; SQJT combined with SHD and JLD combined with SHD were more effective than SHD alone, and the effect values were [OR=1.72, 95%CI=(0.86, 2.58)] and [OR=1.25, 95%CI=(0.42, 2.07)], respectively; SQJT combined with AGI were superior to AGI alone, and the effect value was [OR=1.96, 95%CI=(1.22, 2.71)]. The comparison results of other intervention measures were shown in the figure (**Figure 7**).

##### 3.3.5.2 Cumulative curves under the area chart sort

According to the area ranking diagram under the cumulative curve of clinical efficacy of outcome indicators, JLD combined with RI was the best intervention measure, and the best ranking of various intervention measures was as follows: JLD+RI > SQJT+SHD > SQJT+RI > JLD+Metformin > SQJT+AGI > JLD+GLP-1 > JLD+DPP-IV > SQJT+Metformin > JLD+SHD > JLD+RI > AGI > DPP-IV > GLP-1 > SHD > Metformin > RI (**Figure 7**).

## 4 Discussion

According to the results of this traditional meta-analysis and online meta-analysis, SQJT combined with CWM and JLD combined with CWM have more significant effects on reducing FBG, 2hPG, HbA1c, and the incidence of adverse reactions, as well as by enhancing clinical efficacy, compared with CWM alone. JQJT combined with CWM, TMK combined with CWM, and TMXKP combined with CWM were superior to CWM alone in reducing FBG and 2hPG. After a comprehensive analysis, it was found that SQJT combined with CWM had the most comprehensive effect on the clinical treatment of T2DM.

Clinically, CWM has a very significant hypoglycemic effect in the treatment of T2DM; however, the action pathway is relatively simple. Some CWM inevitably causes certain damage to the heart, liver, kidney, stomach, and other organs, as well as common adverse reactions, such as hypoglycemia, gastrointestinal reactions, allergic reactions, liver and kidney damage, cardiovascular damage, headache, and constipation (71). Studies have shown that some CWM treatments have certain adverse reactions to diabetes and diabetes complications, which may lead to the risk of hypoglycemia in elderly patients with diabetes. Repeated hypoglycemia may lead to cognitive decline and even dementia in elderly patients with diabetes (72). Certain medications, such as sulfonylureas and insulin, can increase the risk of severe hypoglycemia. With the gradual introduction of TCM, the application of TCM in the treatment of diabetes is increasingly extensive. TCM and other natural drugs have strong pharmacological activity, with anti-inflammatory, antioxidant, antidiabetic, and other effects (73). Many types of TCM were included in this study, such as salvia, red peony root, and other blood-activating drugs, which have been proven to maintain blood glucose levels and reduce the content of HbA1c (74). Kudzu root, coptis and rhizome can improve glucose uptake and utilization. Moreover, astragalus combined with Pueraria may regulate the AMPK signaling pathway through IL-6, TNF-α, and other targets to affect insulin resistance, glycogen synthesis, gluconeogenesis, and other processes (75). JQJT is a classic prescription for the treatment of thirst disease in “Bei-ji-qian-jin-yao-fang”. Its components include quercetin, kaempferol, luteolin, β-carotene, and β-sitosterol, which can improve the activity of INS-1 cells stimulated by high glucose, as well as downregulate the relative expression of BAX and PDX-1 mRNA and inhibit the oxidative stress of INS-1 cells. Moreover, apoptosis can be reduced, and the insulin secretion of beta cells can be promoted (76). Previous studies have shown that SQJT can increase the activity of superoxide dismutase (SOD) in the pancreatic tissue of rats, and its hypoglycemic effect may be related to the protection of pancreatic β cells and the restoration of insulin sensitivity in rats. This mechanism may be related to the enhancement of protein kinase B phosphorylation and the upregulation of glucose transporter 2 expression. It plays a hypoglycemic role by promoting glucose uptake and glycogen synthesis (77). Previous studies have found that SQJT can affect vascular factors and play a role in the treatment of T2DM. Through the coordination and mutual influence of the MAPK pathway and VEGF signaling pathway, vascular endothelial growth factor (VEGFA) affects the MAPK signaling pathway by binding with the receptor KDR, thus playing a role in the treatment of T2DM (78). JLD can improve glucose tolerance and reduce blood sugar by regulating the expression of genes and proteins related to the FGF21/AMPK pathway. Studies have shown that the regulation of glucose and lipid metabolism by JLD has a certain correlation with FGF21, which can activate FGF21 and improve the accumulation of lipids in the liver. Furthermore, JLD can further enhance the positive role of FGF21 in regulating glucose and lipid metabolism and in preserving energy homeostasis by activating the FGFR1/β-Klotho receptor complex (79). Schisandra, Mai Dong, and other drugs in TMXKP can enhance insulin sensitivity to a certain extent, but their regulatory effect on blood sugar is limited. The trivalent chromium in TMXKP has a significant effect on reducing blood glucose, inhibiting tyrosine phosphatase, and activating insulin receptor kinase, thus phosphorylating the insulin receptor, reducing insulin resistance and increasing insulin sensitivity. Therefore, it lowers blood sugar levels (80). Moreover, TMK can reduce insulin resistance and improve insulin sensitivity by inhibiting platelet aggregation and thrombin synthesis, thus playing a synergistic role in lowering blood sugar, regulating blood lipids, and improving microcirculation disorders (81). TQJT has a certain effect on lowering blood glucose levels. Gene chip technology has been applied in clinical studies to explore the mechanism of action of TQJT, and the results showed that TQJT can effectively reduce the FBG and triglyceride levels of KKAy mice and improve insulin sensitivity, which may be related to the improvement of glucose utilization *via* the MAPK pathway and GluT-4 upregulation (82). The combination of TCM and CWM is an increasingly widely used diagnosis and treatment method. TCM treatment can improve symptoms and improve clinical efficacy. The combination of TCM with CWM can reduce the dosage of CWM and reduce the clinical side effects caused by CWM. Furthermore, it can delay or reverse the early chronic complications of diabetes and improve quality of life. The combination of TCM and CWM uses modern science and technology, and TCM and CWM complement each other to develop a brand new medical system. Moreover, the combination of disease differentiation and dialysis can better guide the clinical treatment of T2DM.

## 5 Advantages and limitations

There have been few indirect clinical studies on the treatment of T2DM by different TCMs combined with CWM; therefore, the differences in the therapeutic effects of different TCMs combined with CWM are not clear. In this study, network meta-analysis was used to clearly compare the efficacy of different TCMs combined with CWM to guide clinical treatment and provide certain suggestions and aid. However, there were still several shortcomings in this study. (1) The literature included in this study belongs to TCM; inevitably, most of the studies were domestic studies, with few relevant foreign studies. (2) As limited by statistical methods, some of the relevant methods that were included in the study were not optimal, and the quality was not good. (3) The intervention time was not long enough for the included studies, the efficacy in the future for a long period of time was not clear. (4) The sample size that was included in the study was not large enough. When considering the above mentioned aspects, we will focus on large-sample, multicenter, double-blind, and other high-quality clinical studies in the future to improve our research conclusions, with a view to providing more authoritative and instructive suggestions for the clinical treatment of T2DM.

## 6 Conclusion

Through the combination of this traditional meta-analysis and network meta-analysis, it was concluded that different TCMs combined with CWM had more significant clinical efficacy and safety in the treatment of T2DM compared with CWM alone, among which SQJT combined with CWM may be the best intervention measure, SQJT combined with SHD and Metformin is the best clinical method to reduce blood glucose content, and JLD combined with RI has the best clinical effect. The order of the best possible intervention measures for different outcome indicators was also obtained. The aim of the study outcomes is to provide some suggestions and help for clinical applications.

## Data availability statement

The original contributions presented in the study are included in the article/supplementary material. Further inquiries can be directed to the corresponding author.

## Author contributions

KM and LZ were involved in study design, data collection, analysis, and manuscript revision. KM and ML contribute to data collection, data interpretation and manuscript revision. CT and CY participated in data analysis, data interpretation, manuscript drafting and revision, YZ and JZ were responsible for the concise and unobstructed language expression of the article and graphic production. KM is the guarantor of this work, and ML is the corresponding author of this paper, providing valuable suggestions for the overall framework and content construction of this paper. All authors contributed to the article and approved the submitted version.
